# Supplementary material for: Mesothelin Expression Is Not Associated with the Presence of Cancer Stem Cell Markers SOX2 and ALDH1 in Ovarian Cancer
Source: Int J Mol Sci. 2022 Jan 18;23(3):1016. doi: 10.3390/ijms23031016 (PMC8834752; doi:10.3390/ijms23031016)
Supplement: Supplementary file 1 [file ijms-23-01016-s001.zip › ijms-1515361-supplementary.pdf]

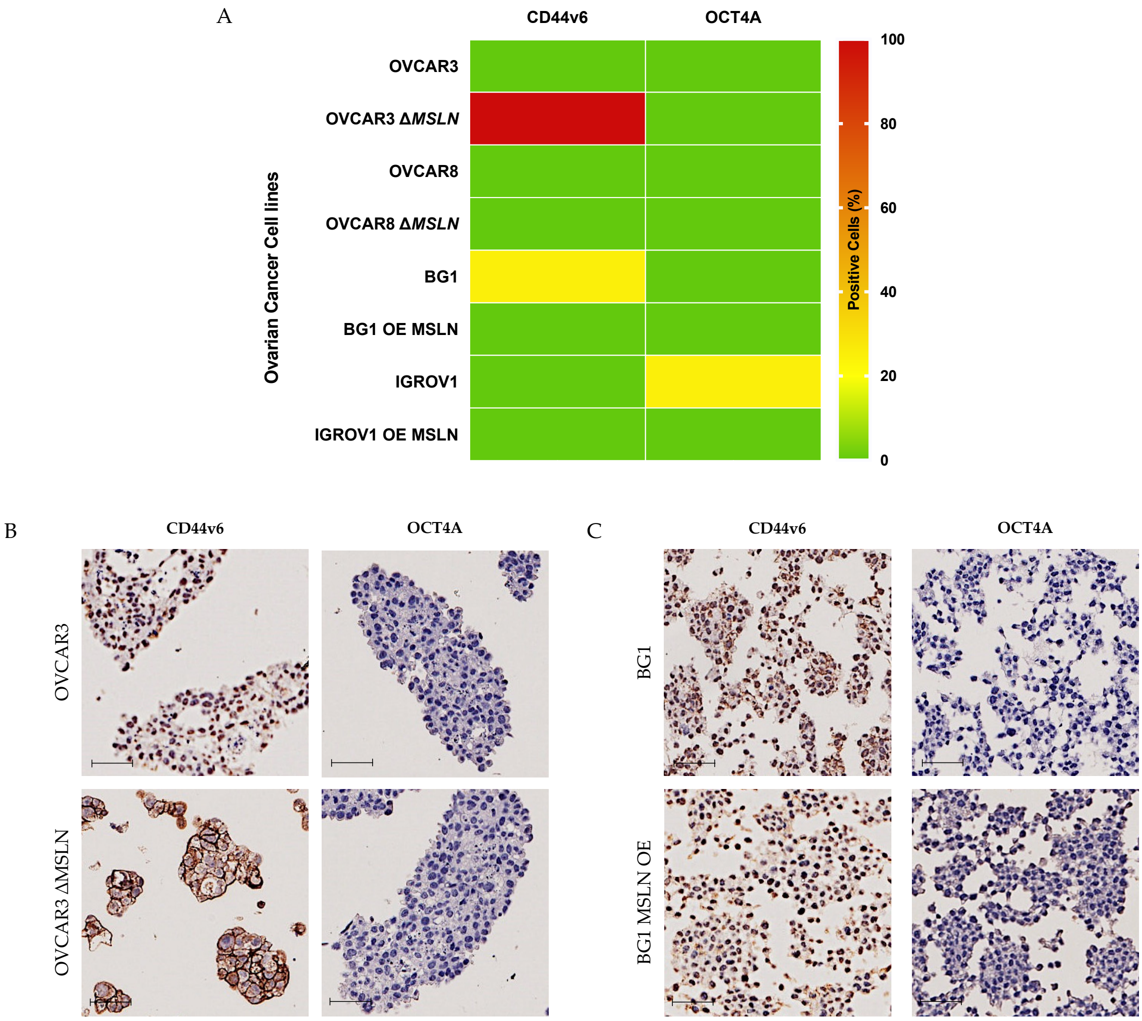

Figure S1. CSC markers CD44v6 and OCT4A expression in ovarian cancer (OC) cell lines with respective mesothelin (MSLN) knockout (Δ) or overexpression (OE). (**A**) Heat map showing the membrane expression profile of CD44v6 and OCT4A in a panel of eight OC cell lines cultured under 3D conditions. Colour key represents the percentage of positive cells for each marker. (**B**,**C**) Representative immunohistochemistry (IHC) images for CD44v6 (membrane) and OCT4A expression patterns in OVCAR3 and OVCAR3 Δ*MSLN* cells and in BG1 and BG1 MSLN OE cells (**B**, **C**). Scale bar: 50 µm.
